# P-1850. Enhancing Communication of Antimicrobial Side Effects for OPAT Patients at Discharge

**DOI:** 10.1093/ofid/ofaf695.2019

**Published:** 2026-01-11

**Authors:** Bojan Lazarevic, Isaac Daudelin, Jordan Feingold-Link, Nehar Damle, Caroline Brugger, Rachel Carr, Devin Weber, Daniel Taupin

**Affiliations:** Thomas Jefferson University Hospital, Philadelphia, PA; Thomas Jefferson University, Philadelphia, Pennsylvania; Thomas Jefferson University Hospital, Philadelphia, PA; Thomas Jefferson University Hospital, Philadelphia, PA; Thomas Jefferson University Hospital, Philadelphia, PA; Thomas Jefferson University Hospital, Philadelphia, PA; Department of Medicine, Division of Infectious Diseases, Sidney Kimmel Medical College at Thomas Jefferson University, Philadelphia, PA; Thomas Jefferson University Hospital, Philadelphia, PA

## Abstract

**Background:**

Patients discharged on antimicrobial therapy often receive insufficient information about side effects requiring medical attention. This project aimed to implement an electronic health record (EHR)-based intervention to improve provider documentation of antimicrobial side effects in the After Visit Summary (AVS) for patients enrolled in an Outpatient Parenteral Antimicrobial Therapy (OPAT) program.

**Figure 1:**
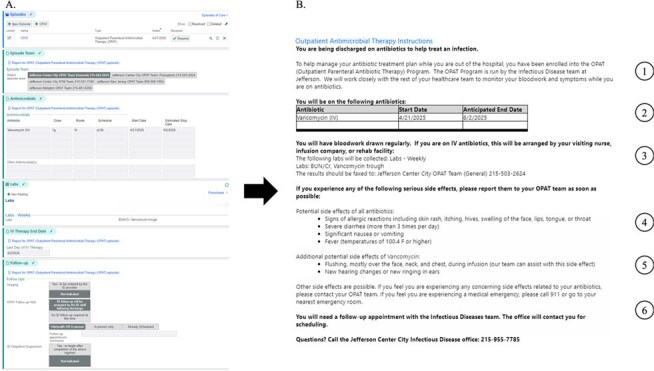
Intervention Overview. (A) Infectious disease provider view of intervention in Epic. (B) Annotated example of an After Visit Summary printout with antimicrobial information pulled from intervention. 1 – Information about OPAT enrollment. 2 – Antimicrobial(s) and therapy end dates. 3 – Required laboratory monitoring while on therapy. 4 – Generic antimicrobial side effects included in every set of OPAT discharge instructions. 5 – Specific antimicrobial side effects included by infectious disease provider based on antimicrobial class. 6 – Information on whom to contact in case of questions or emergencies.

**Figure 2:**
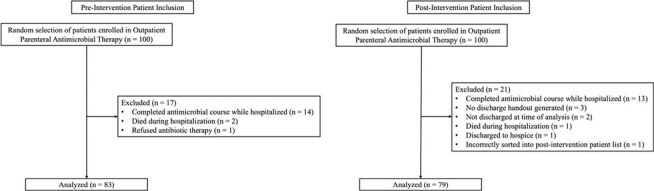
Pre-Intervention and Post-Intervention Patient Inclusion Flowcharts.

**Methods:**

Using Plan-Do-Study-Act (PDSA) methodology, a multidisciplinary team at a large, urban academic center identified opportunities to improve discharge communication about antimicrobial side effects for OPAT patients. A provider survey informed the intervention design. We created an EHR-integrated tool in Epic to allow Infectious Disease (ID) providers to add standardized antimicrobial recommendations into a navigator that automatically transfers to the AVS (Figure 1). We conducted pre- and post-intervention chart reviews (n=200) to assess the intervention. Measures included the presence of the following in the AVS: antimicrobial recommendations with therapy end dates, generic and specific antimicrobial side effects, and the ID office number. We conducted Chi-squared analyses to assess statistical significance.

**Figure:**
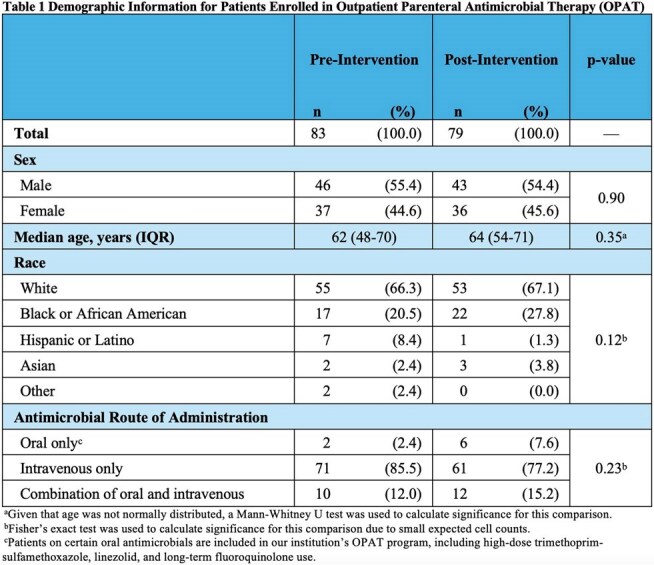

**Figure:**
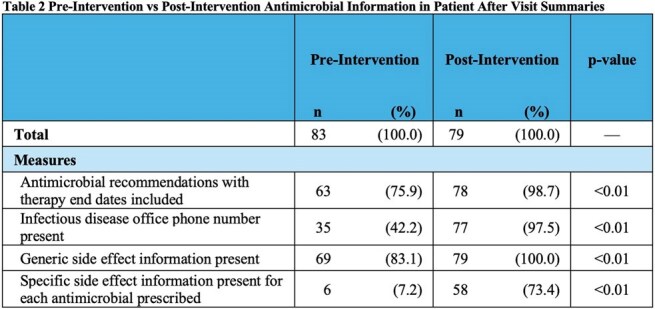

**Results:**

After excluding ineligible patients, 83 patients were included in the pre-intervention analysis and 79 in the post-intervention analysis (Figure 2). Demographic information is shown in Table 1. There were no significant differences in distribution of sex, age, race, or route of antimicrobial administration among the two groups. Compared to pre-intervention AVSs, post-intervention AVSs were more likely to include antimicrobial recommendations with therapy end dates (75.9% vs 98.7%, p< 0.01), ID office phone number (42.2% vs 97.5%, p< 0.01), generic antimicrobial side effects (83.1% vs 100.0%, p< 0.01), and specific antimicrobial side effects (7.2% vs 73.4%, p< 0.01) (Table 2).

**Conclusion:**

An EHR-based intervention significantly improved the documentation of antimicrobial information in OPAT patient AVSs. This intervention addressed a critical gap in communication during care transitions. A key limitation is that AVS documentation doesn't guarantee patient education; this can be assessed in future PDSA cycles with patient surveys.

**Disclosures:**

All Authors: No reported disclosures

